# Highlights on Future Treatments of IPF: Clues and Pitfalls

**DOI:** 10.3390/ijms25158392

**Published:** 2024-08-01

**Authors:** Alessandro Libra, Enrico Sciacca, Giuseppe Muscato, Gianluca Sambataro, Lucia Spicuzza, Carlo Vancheri

**Affiliations:** 1 Department of Clinical and Experimental Medicine, Regional Referral Center for Rare Lung Disease, Policlinico “G. Rodolico-San Marco”, University of Catania, 95123 Catania, CT, Italy; alessandrolibra@outlook.it (A.L.); esciacca29@gmail.com (E.S.); gpp.muscato@gmail.com (G.M.); lucia.spicuzza@unict.it (L.S.); 2 Artroreuma s.r.l., Rheumatology Outpatient Clinic, 95030 Mascalucia, CT, Italy; dottorsambataro@gmail.com

**Keywords:** idiopathic pulmonary fibrosis, IPF, pathogenesis, molecular mechanism, therapy, clinical trials, target therapies, future perspective

## Abstract

Idiopathic pulmonary fibrosis (IPF) is an interstitial lung disease characterized by irreversible scarring of lung tissue, leading to death. Despite recent advancements in understanding its pathophysiology, IPF remains elusive, and therapeutic options are limited and non-curative. This review aims to synthesize the latest research developments, focusing on the molecular mechanisms driving the disease and on the related emerging treatments. Unfortunately, several phase 2 studies showing promising preliminary results did not meet the primary endpoints in the subsequent phase 3, underlying the complexity of the disease and the need for new integrated endpoints. IPF remains a challenging condition with a complex interplay of genetic, epigenetic, and pathophysiological factors. Ongoing research into the molecular keystones of IPF is critical for the development of targeted therapies that could potentially stop the progression of the disease. Future directions include personalized medicine approaches, artificial intelligence integration, growth in genetic insights, and novel drug targets.

## 1. Introduction

Idiopathic pulmonary fibrosis (IPF) is a chronic interstitial lung disease (ILD) characterized by irreversible scarring of lung tissue and destruction of lung architecture, exhibiting radiological and histological characteristics consistent with usual interstitial pneumonia [1]. IPF represents, as a form of idiopathic interstitial pneumonia, a subset of fibrotic lung disorders that lack a discernible cause, making its etiology elusive and understanding of its pathogenesis challenging.

The pathologic hallmark of IPF is the aberrant deposition of collagen and other extracellular matrix components within lung parenchyma, leading to impaired gas exchange and altered lung function [2]. This condition predominantly affects individuals in their sixth decade of life, presenting an insidious onset, progressive nature, and limited therapeutic options. Patients with IPF report symptoms like dyspnea, dry cough, and fatigue [1]. However, the non-specific nature of these symptoms poses significant challenges in early diagnosis; consequently, IPF is frequently diagnosed at advanced stages, limiting the effectiveness of therapeutic interventions [3]. Unraveling the pathogenesis of IPF has been a subject of intensive research, revealing complex interactions among genetic predisposition, environmental factors, and dysregulated host responses. Alveolar epithelial cell (AEC) injury, abnormal wound healing responses, and dysregulation of fibroblast activation are key cellular events implicated in the initiation and perpetuation of the fibrotic cascade [2,4]. A deeper understanding of the molecular and cellular mechanisms involved in IPF is essential for the development of targeted therapies that can stop or reverse the progression of this devastating disease. Despite advancements in the understanding of IPF pathobiology, therapeutic options remain limited. Antifibrotic agents, such as pirfenidone and nintedanib, have emerged as the mainstay of treatment, aiming to slow disease progression. However, these drugs are not curative, and the need for novel therapeutic modalities that address the underlying pathogenic processes is evident. The complex and multifaceted nature of IPF needs more research efforts to fully elucidate its underlying mechanisms. The absence of a definitive cure has encouraged investigations in the field of IPF. However, a significant number of trials, particularly those in phase III, have failed to achieve the primary endpoint [5].

This review aims to contribute to the collective knowledge that will pave the way for innovative approaches to treatment and to provide a deeper understanding of the latest developments in the pathogenesis of IPF, highlighting recent progress in pharmaceutical interventions for IPF.

## 2. Genetics, Risk Factors, and Epigenetic Changes in the Pathogenesis of IPF

Genetic factors play a fundamental role in the development of IPF. Genetic variants associated with IPF are classified into two categories: single nucleotide polymorphisms (SNPs) found in the general population with an allele frequency greater than 1% and rare variants with an allele frequency lower than 1%, usually not present in the general population [6]. The development of next-generation sequencing (NGS) technologies has allowed a deeper exploration of the contribution of genomic variants to IPF development, focusing the attention on those variants involving two different biological pathways, namely telomere maintenance and surfactant metabolism [7]. Telomeres are specialized structures at the ends of chromosomes with the function of protecting genome integrity and preventing end-to-end chromosomal degradation and fusion [8]. During DNA replication, the extreme ends of chromosomes cannot be copied, leading to progressive telomere shortening with each cell division. Telomerases are enzymes that add repeated sequences to the ends of chromosomes to compensate for this telomere shortening. However, these enzymes have limited activity and gradually lose their function, ultimately resulting in cellular senescence and apoptosis [9]. Telomerases involve a catalytic subunit, telomerase reverse transcriptase (TERT), and the RNA component of telomerase (TERC). Genetic defects altering the activity of TERT and TERC were the first described and the most common in IPF patients [10,11]. With the development of NGS technology, numerous genetic variants contributing to the pathogenesis of IPF have been identified, showing that patients with mutations in telomere-related genes have shortened telomeres that may lead to an early onset of the disease [7]. Pulmonary surfactant is a mixture of lipids and proteins produced by type II AEC. In addition to playing a role in host defense and modulating the immune response, its primary function is to reduce the surface tension of alveoli, preventing their collapse. Mutations in these genes predispose individuals to a wide range of fibrotic lung diseases [12,13,14,15]. The genetic variant most strongly associated with IPF susceptibility is a SNP located in the promoter region of the MUC5B gene, identified as rs35705950 [16,17]. MUC5B encodes mucin 5B, a component of the mucus on the surface of the bronchial mucosa, and is associated with the failure of alveolar repair, regeneration mechanisms, and mucociliary dysfunction [18,19]. Genetic variants of MUC5B cause its overexpression in bronchoalveolar epithelium and represent one of the main genetic risk factors for both familial and sporadic IPF, although the precise mechanism involved in disease induction is not yet clear [20,21]. Cigarette smoke is one of the primary risk factors for chronic respiratory diseases, including IPF [22]. It can cause damage to all types of lung cells, but it particularly harms AEC, triggering the fibrogenic process [23]. Cigarette smoke increases the risk of IPF, with smokers having a 60% higher risk [24]. In addition to cigarette smoke, particulate matter, fibers, and dust are major environmental risk factors contributing to the onset of IPF [25]. An increased incidence of the disease was observed in individuals exposed to inorganic dust, chemical fumes, and other pollutants [26]. These substances, following prolonged exposure, result in epithelial damage and oxidative stress, leading to senescence of AEC type II with consequent fibrosis [27,28]. These risk factors can induce epigenetic alterations, such as DNA methylation, histone modification, and non-coding RNA gene silencing, which play a key role in the development of pulmonary fibrosis [29,30,31]

## 3. Overview of Current Pathogenic Hypothesis

IPF is characterized by the excessive production and disorganized deposition of extracellular matrix (ECM) components, resulting in irreversible architectural distortion and loss of organ function [32]. One crucial player in the multifaceted pathogenesis of IPF is transforming growth factor (TGF)-β. Released in response to epithelial cell injury, TGF-β acts as a central pro-fibrotic growth factor, driving the progression of pulmonary fibrosis [33]. Its multifunctional nature stimulates the proliferation and differentiation of epithelial cells and fibroblasts, activates myofibroblasts to generate ECM, catalyzes epithelial-mesenchymal transition (EMT), expedites epithelial apoptosis and cell migration, and induces the production of connective tissue growth factor (CTGF) and other mediators [26,34]. Insulin-like growth factor (IGF) also contributes significantly to the progression of pulmonary fibrosis [35,36]. IGF-1 plays a role in mediating various biological functions, including fibroblast proliferation, migration, and differentiation. This enhances the ability of fibroblasts to synthesize fibronectin and collagen, ultimately leading to increased ECM deposition [37,38].

CTGF, as a cysteine-rich stromal cell protein, influences numerous biological processes, such as cell proliferation, differentiation, adhesion, and angiogenesis [39]. It acts as a primary mediator of TGF-β-induced pulmonary fibrosis, directing tissue regeneration and pathological fibrosis formation through ECM deposition, fibroblast proliferation, and matrix generation [40,41]. Matrix metalloproteinases (MMPs), including MMP-3, MMP-7, and MMP-8, actively contribute to pulmonary fibrosis by regulating EMT and influencing abnormal repair processes [42,43]. MMP-7, in particular, is elevated in both human IPF and mouse fibrosis models, and higher levels are associated with an increased risk of mortality and disease progression [44]. Exosomes, phospholipid bilayer membranous vesicles, also play a significant role in the pathogenesis of IPF [45]. Continuously secreted by various cell types, they transport biologically active substances, such as proteins, lipids, and genetic material. In the lungs, alveolar and bronchial epithelial cells primarily generate exosomes, which activate fibroblasts, stimulate their differentiation into myofibroblasts, and catalyze excessive ECM component deposition [46]. Aging further contributes to IPF by depleting type 2 AEC, impairing the alveoli’s ability to repair injury. IPF lung tissue exhibits several characteristics of aging lungs, including cellular senescence, telomere shortening, mitochondrial and lysosomal/autophagy dysfunction, and epigenetic changes [14,15].

Tumor necrosis factor-alpha (TNF-α), a pro-inflammatory cytokine, significantly contributes to the recruitment and activation of immune cells, exacerbating tissue damage and fibrosis. TNF-α directly stimulates pulmonary fibroblasts, increasing the production of extracellular matrix and fibrogenic growth factors, creating a pro-fibrotic environment [47]. Interleukins (IL), particularly IL-1β, IL-6, and IL-13, promote fibroblast proliferation and collagen deposition, enhancing extracellular matrix remodeling and scarring of lung tissue [48].

**Figure 1:**
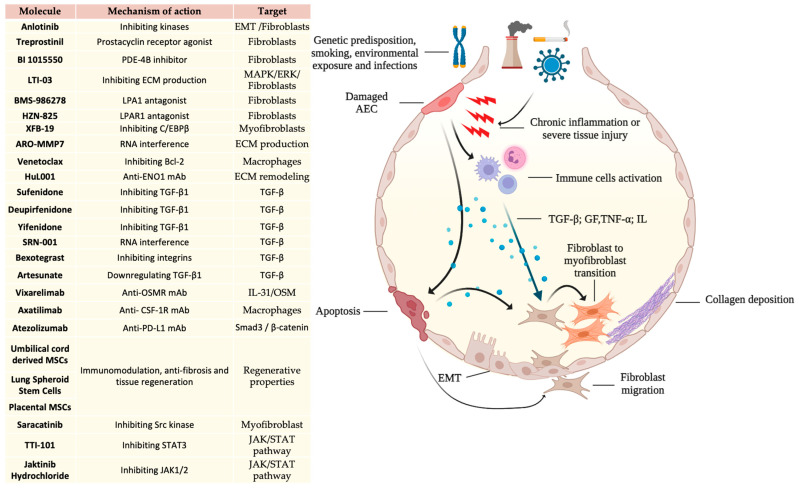
Overview of the most significant pathogenic pathways involved in the pathogenesis of idiopathic pulmonary fibrosis (IPF) and molecules currently under clinical investigation and their mechanisms of action within the pathways implicated in IPF pathogenesis. Legend: AEC: alveolar epithelial cells; EMT: epithelial-mesenchymal transition; PDE-4B: phosphodiesterase-4B; ECM: extracellular matrix; MAPK/ERK: mitogen-activated protein kinase/extracellular signal-regulated kinase; LPA1: lysophosphatidic acid receptor 1; C/EBPβ: CCAAT/enhancer binding protein beta; mAb: monoclonal antibody; Bcl-2: B-cell lymphoma 2; TGF-β: transforming growth factor beta; GF: growth factor; TNF-α: tumor necrosis factor alpha; IL: interleukin; OSMR: oncostatin M receptor; CSF-1R: colony stimulating factor 1 receptor; PD-L1: programmed death—ligand 1; Smad3: SMAD family member 3; MSCs: mesenchymal stem cells; STAT3: signal transducer and activator of transcription 3; JAK1/2: Janus kinase 1/2.

In summary, the intricate pathogenesis of IPF (Figure 1) involves a dynamic interplay of factors, including TGF-β, IGF, CTGF, MMPs, exosomes, TNF-α, and interleukins, all contributing to the excessive production and deposition of ECM components, ultimately resulting in irreversible architectural distortion and loss of organ function. Understanding these mechanisms provides potential avenues for therapeutic interventions in IPF.

## 4. Future Therapeutic Perspectives Based on Current Pathogenic Knowledge

Current approved anti-fibrotic drugs, pirfenidone and nintedanib, target multiple known aspects of IPF. Pirfenidone reduces TGF-β expression and activation, mitigating fibroblast activation and collagen synthesis [49]. Pirfenidone also exhibits anti-inflammatory properties by suppressing pro-inflammatory cytokines and chemokines, reducing lung inflammation [50].

Nintedanib, a tyrosine kinase inhibitor, targets receptors involved in fibrosis such as vascular endothelial growth factor receptor (VEGFR), fibroblast growth factor receptor (FGFR), and platelet-derived growth factor receptor (PDGFR) [51,52].

Several molecules are under investigation in various clinical trial in different phases. The main clinical trials related to ongoing studies for IPF therapy are summarized in Table 1.

### 4.1. The Role of Fibroblasts

In healthy lung tissue, fibroblasts play a crucial role in regulating wound healing and tissue repair. They are primarily responsible for synthesizing and remodeling the ECM, thus ensuring tissue homeostasis [32]. In the context of IPF, recurrent injurious triggers, along with a compromised function of the alveolar epithelium, change the cytokine balance within lung tissue. The alteration of the tissue microenvironment is characterized by elevated levels of profibrotic molecules such as platelet-derived growth factor (PDGF), fibroblast growth factor (FGF), and TGF-β [53]. This promotes the transformation of fibroblasts into myofibroblasts (FMT) and the inhibition of the subsequent programmed cell death of these cells [54]. This process is marked by the proliferation of resident mesenchymal cells, the attraction of circulating fibrocytes, and the stimulation of EMT [55]. Emerging evidence suggests that epithelial cells undergoing EMT may also contribute to the pool of activated myofibroblasts in IPF [53]. Activated fibroblasts and myofibroblasts in IPF are prolific producers of ECM components, particularly collagen. This uncontrolled deposition of ECM results in lung stiffening and impaired gas exchange, contributing to the characteristic decline in lung function seen in IPF patients [56].

Tyrosine kinase and its receptor are also involved in regulating cell growth and fibroblast behavior. In 2014, FDA approved nintedanib, a tyrosine kinase receptor inhibitor, for the treatment of IPF [57]. Anlotinib represents a novel class of small-molecule, multi-target tyrosine kinase inhibitors. Its mechanism of action is based on the suppression of the activity of VEGFR, PDGFR, FGFR, and various other kinases. It received regulatory approval in China in 2018 and has gained significant utilization in anti-angiogenesis therapy for lung cancer patients in recent years [58]. In addition, preclinical studies have shown that anlotinib can inhibit the proliferation of AEC-induced EMT and pulmonary fibroblasts by inhibiting the TGFβ-1 signaling pathway. In addition, anlotinib inhibits pulmonary fibrosis by down-regulating the poly(rC)-binding protein 3 (PCBP3) expression, reducing 6-phosphofructo-2-kinase/fructose-2,6-bisphosphatase-3 (PFKFB3) translation, and inhibiting glycolysis of fibroblasts [59,60]. A phase 2–3 trial (NCT05828953) is recruiting patients with IPF/progressive fibrosing interstitial lung diseases (PF-ILDs) diagnoses to evaluate the effectiveness of anlotinib hydrochloride capsules in reducing forced vital capacity (FVC) decline.

Treprostinil is a full prostacyclin receptor (IP) agonist that has high affinity for prostaglandin E receptor 2 (EP2) and the prostaglandin D receptor 1 (DP1). Binding to and activating EP2, IP, DP1, and peroxisome proliferator-activated receptor β (PPARβ) receptors lead to a range of antifibrotic effects [61]. When EP2, IP, and DP1 receptors are activated, they cause vasodilation and help reduce fibroblast activity, proliferation, collagen deposition, and inflammation. Specifically, EP2 activation inhibits the transformation of fibroblasts into myofibroblasts and reduces collagen overproduction [62,63], while DP1 activation decreases the recruitment of inflammatory cells and the synthesis of the extracellular matrix [64]. Additionally, activating the PPARβ receptor suppresses fibroblast proliferation [65]. Together, these actions promote vasodilation, minimize vascular remodeling, and reduce fibrosis. The INCREASE trial was a randomized, placebo-controlled, phase 3 study that assessed the effects of inhaled treprostinil in patients with ILD and associated pulmonary hypertension. The trial found that treprostinil significantly improved exercise capacity from baseline to week 16, as measured by the 6-min walk test, compared to placebo. Additionally, the study showed improvements in forced vital capacity (FVC) and reduced exacerbations of the underlying lung disease [66]. Beyond achieving the primary endpoint (6-min walk distance) and secondary endpoints, a post hoc analysis revealed significant improvements in FVC for subjects with PH-ILD treated with inhaled treprostinil [67]. These results, along with preclinical evidence of treprostinil’s antifibrotic activity, suggest that inhaled treprostinil may be a treatment option for patients with IPF. Currently, the TETON program includes two phase 3 randomized, double-blind, placebo-controlled studies (NCT04708782, NCT05255991) that are investigating the efficacy and safety of inhaled treprostinil in idiopathic pulmonary fibrosis [68].

Phosphodiesterases (PDEs) are the main superfamily of enzymes responsible for degrading the secondary messengers cyclic adenosine monophosphate (cAMP) and cyclic guanosine monophosphate (cGMP). Inhibiting these enzymes results in a decrease in prostaglandin E2 levels, which plays a crucial role in fibroblast function. [69] BI 1015550, a phosphodiesterase-4B (PDE-4B) inhibitor, has shown potential in preclinical models to inhibit TGFβ1-induced myofibroblast transformation and extracellular matrix deposition [70]. A phase 2 double-blind, placebo-controlled, parallel-design trial showed that patients treated with BI 1015550 had a smaller median decline, or a slight improvement, in FVC at 12 weeks compared to the placebo group (median difference of 62–84 mL) [71]. The FIBRONEER phase 3 program is currently recruiting globally to assess BI 1015550 in IPF (NCT05321069).

Caveolin-1 (Cav-1) is another crucial regulator of TGF-β signaling. TGF-β receptors internalized through Cav-1 undergo rapid degradation, reducing TGF-β signaling. Cav-1 deficiency results in increased collagen gene expression in human lung fibroblasts. Restoration of Cav-1 function using a cell-permeable peptide carrier for a bioactive Cav-1 fragment has shown to reduce fibrosis, indicating Cav-1 as a potential therapeutic target for pulmonary fibrosis [72]. Cav-1 influences the mitogen-activated protein kinases/ extracellular signal-regulated kinases (MAPK/ERK) signaling pathway in lung fibroblasts. A decrease in Cav-1 expression leads to increased MAPK/ERK activation and collagen expression. This shows that Cav-1 plays a role in a branched signaling pathway that regulates collagen expression in lung fibroblasts, damaged during fibrotic process [72]. A marked reduction in Cav-1 expression is observed in lung tissues and pulmonary fibroblasts from IPF patients. Cav-1’s ability to suppress TGF-β1-induced ECM production through the regulation of the c-Jun N-terminal kinases (JNK) pathway has been highlighted [73]. Based on this evidence, the LTI-03 (NCT05954988) study will investigate the caveolin-1-scaffolding-protein-derived Peptide, recruiting IPF patients for a phase 1 clinical trial.

Lysophosphatidic acid (LPA) also plays a role in the pathogenesis of IPF, particularly through its interactions with fibroblasts. Recent research has revealed significant insights into how LPA and its related pathways contribute to IPF. LPA signaling occurs through receptors found in various cells, including AEC, vascular endothelial cells, and fibroblasts. LPA receptor inhibitors and metabolic enzymes involved in LPA formation may represent potential targets for treating IPF [74]. In this context, the LPA1 receptor has emerged as a promising therapeutic target. A novel LPA1 antagonist, BMS-986278, has shown efficacy in preclinical studies by inhibiting LPA-stimulated responses in primary human lung fibroblasts and showing antifibrotic activity in animal models. This finding suggests that targeting LPA1 could slow the progression of IPF [75]. In 2023, a phase 3 study (NCT06003426) started recruiting participants with IPF to test BMS-986278. Phase 2 results were encouraging, showing a significantly slowed rate of FVC decline [76]. Following the lead of BMS-986278, a LPAR1 antagonist (NCT05032066) coded as HZN-825 will be investigated for its potential in treating IPF and systemic sclerosis (SSc).

The enzyme autotaxin, which generates most extracellular LPA, is also implicated in the development of lung fibrosis. Elevated autotaxin expression was found in the lungs of IPF patients, and the inhibition of this enzyme limits the development of pulmonary fibrosis in animal models [77]. This recent finding places autotaxin as an interesting new target for IPF and other fibrotic diseases [78]. Unfortunately, the ISABELA 1–2 trials (NCT03711162-NCT03733444) that tested ziritaxestat, an autotaxin inhibitor, in patients with IPF did not improve clinical outcomes compared with placebo [79].

The chronic activation and prolonged presence of myofibroblasts within tissues are related to the progression of fibrosis [80]. CCAAT/enhancer-binding protein beta (C/EBPβ) is emerging as a critical factor in the pathogenesis of IPF, particularly through its interactions with fibroblasts and effect on myofibroblast differentiation. Studies, such as the one conducted by Ding et al., reveal that C/EBPβ acetylation is significantly elevated in IPF, especially within fibroblast foci. This acetylation correlates with an increase in α-smooth muscle actin (α-SMA) and collagen-I, markers indicative of pulmonary fibrosis. This finding underscores the involvement of acetylated C/EBPβ in the development of pulmonary fibrosis and suggests a potential target for therapeutic intervention [80,81]. XFB19 (NCT05361733) is a first-in-class synthetic tetra-peptide (Acetyl-Lys-D-Ala-D-Val-Asp-NH2) that inhibits the activation of the human C/EBPβ, thus mediating the activation of lung myofibroblasts.

MMP7, also known as matrilysin, is a member of the matrix metalloproteinase family of enzymes that are involved in the degradation and remodeling of ECM components in the lung tissue, expressed by lung epithelial cells, mononuclear phagocytes, and fibrocytes [82]. Moreover, increased levels of MMP7 were found in sputum and bronchoalveolar lavage (BAL) of patients with IPF, suggesting a plausible role in fibrogenesis and a possible role as a biomarker [83,84]. A clinical trial is going to test ARO-MMP7, an RNA interference (RNAi), as a therapeutic candidate [85] that targets MMP7 expression, aiming at, as the primary outcome, the changes in MMP7 concentration in BAL (NCT05537025).

### 4.2. The Role of Macrophages

It is known that macrophages contribute to the maintenance of tissue homeostasis, immune regulation, and pathogen clearance [86]. Within the lungs, there are different types of macrophages, such as alveolar macrophages (AMs) and interstitial macrophages (IMs), each with different characteristics, responsible for specific responses to different signals [87]. Macrophages are fundamental for the regulation of lung immunity, acting as a major component of the innate immune system and providing a link with adaptive immunity. They play a significant role in responding to toxic exposures and environmental challenges [88]. Their heterogeneity and plasticity enable them to efficiently respond to various cytokines and microbial signals [89]. Their motility allows them to migrate within lung tissues, adapting their function to their environment and playing roles ranging from phagocytic scavengers to microbicidal effectors [90]. Apoptosis resistance in monocyte-derived macrophages (MDMs) is a notable feature of IPF. MDMs isolated from the BAL of patients with IPF, exhibit increased mitochondrial biogenesis partly due to heightened expression of peroxisome proliferator-activated receptor gamma coactivator 1-alpha (PGC-1α), a coactivator regulating mitochondrial dynamics. This contributes to apoptosis resistance in MDMs [91]. Elevation of mitochondrial Bcl-2 is linked instead to interactions with carnitine palmitoyltransferase 1a (Cpt1a), which binds to Bcl-2’s BH3 domain, anchoring it in the mitochondria to reduce apoptosis. Consequently, modulating the interaction between Cpt1a and Bcl-2 in macrophages could influence fibrotic remodeling and apoptosis resistance [91]. Based on this preclinical evidence, a new drug, venetoclax, a new Bcl-2 inhibitor and BH3 domain blocker, has been shown to induce apoptosis of MDMs and reverse established fibrosis [92,93]. The NCT05976217 study is recruiting to test venetoclax in an early phase 1 clinical trial. ENO1, also known as alpha-enolase, is a multifunctional glycolytic enzyme involved in various physiological processes. It plays a crucial role in glycolysis, the metabolic pathway that converts glucose into pyruvate, generating ATP and NADH in the process. ENO1 is also involved in other non-glycolytic functions, such as cell signaling, transcriptional regulation, and cell migration [94]. In addition, ENO1 interacts with human plasminogen, suggesting its involvement in the regulation of fibrinolysis and ECM remodeling [95]. HuL001 is an anti-ENO1 monoclonal antibody that will be investigated in a phase 1 clinical trial (NCT04540770).

### 4.3. The Role of TGF-β

Repeated, chronic damage to AEC causes an elevated secretion of profibrotic cytokines and chemokines [96]. The increased release of fibrogenic signaling molecules, like TGF-β, promote recruitment, propagation, and transformation of fibroblasts into myofibroblasts. TGF-β increases collagen synthesis, exerting its influence on the primary collagen-producing cells [27,28]. Additionally, TGF-β stimulates production of other fibrogenic molecules, such as CTGF, FGF, IGF, and PDGF. TGF-β is not only a potent stimulator of ECM production; it is also known as the most potent attractant for immune cells, encompassing monocytes and macrophages [97]. Pirfenidone, one of two antifibrotic drugs approved for treatment of IPF, plays a role as an antifibrotic agent exhibiting proficient inhibition of fibronectin and α-SMA expression, both pivotal factors in the EMT transition induced by TGF-β [98,99,100]. Sufenidone (NCT06125327), deupirfenidone (NCT05321420), and yifenidone (NCT05060822) are three molecules with a chemical structural similar to pirfenidone. While sufenidone seems to have a protective effect on pulmonary fibrosis in mouse models [101], deupirfenidone and yifenidone are derived forms of pirfenidone designed to attenuate the rate of drug metabolism, resulting in a differentiated pharmacokinetic profile that maintains the efficacy of pirfenidone with a potential improvement of tolerability [102,103].

SRN-001 (NCT05984992) represents an innovative small interfering RNA (siRNA) under development for the treatment of fibrosis (phase 1). Amphiregulin (AREG), a growth factor intricately involved in fibroblast proliferation and differentiation into myofibroblasts, stands as a pivotal player in the characteristic fibrosis observed in lung tissues [104]. AREG emerges as a downstream gene excessively expressed by TGF-β during fibrosis, thereby instigating FMT [105]. Through the mechanism of RNA interference (RNAi), SRN-001 is designed to attenuate the production of amphiregulin, aiming to modulate the fibrotic processes.

Integrins constitute an extensive group of transmembrane glycoprotein receptors originally recognized for their roles in cell adhesion and maintenance of tissue integrity. Among these, integrin αVβ6 plays a crucial role in the activation of latent TGF-β, exhibiting a mechanism that enables control of its activity [106]. Bexotegrast (NCT06097260), inhibiting integrins αVβ1 and αVβ6, which in turn blocks the activation of TGF-β [107,108], is currently in a phase 2 trial aimed to evaluate efficacy and safety.

Artesunate is a semi-synthetic artemisinin, widely used in clinical antimalarial treatment. Several studies have demonstrated the role of artesunate in downregulating the expression of profibrotic mediators, such as TGF-β1, in rats with bleomycin-induced pulmonary fibrosis [109,110]. According to these concepts, a phase 1 study evaluating the safety and tolerability of artesunate with escalating doses is currently ongoing (NCT05988463).

### 4.4. New Therapeutic Strategies: Monoclonal Antibody

Current medical practices have undergone a transformative shift towards tailored treatments addressed to individualized disease characteristics. Monoclonal antibodies (mAbs) serve as an illustration of personalized therapeutics, made possible through advances in our understanding of immunology, molecular biology, and biochemistry.

CTGF’s role in regulating myofibroblast activation, ECM deposition, and fibrotic remodeling through TGF-β downstream signaling is considered a key factor of IPF pathogenesis [111,112,113]. The PRAISE phase 2 trial investigated the effects of pamrevlumab, a recombinant human antibody against CTGF, on the decline in the percentage of predicted FVC at week 48. Pamrevlumab reduced the decline in the percentage of predicted FVC, and positive treatment outcomes were evidenced, such as the improvement of some radiological findings (quantitative lung fibrosis score at HRCT) and symptoms. The promising results from the phase 2 trial led to the initiation of a phase 3 program consisting of two identical trials (ZEPHYRUS I and II), (NCT03955146 and NCT04419558). Unfortunately, this program faced premature termination in June 2023, as the ZEPHYRUS I study did not meet the primary endpoint. Vixarelimab is another human monoclonal antibody that targets oncostatin M receptor beta (OSMRβ), which mediates signaling of interleukin-31 (IL-31) and oncostatin M (OSM). The rationale for a clinical trial with this antibody is based on the evidence that loss of IL-31 signaling attenuates bleomycin-induced pulmonary fibrosis, while elevated expression levels of OSM have been noted in various inflammatory conditions, including those accompanied by fibrotic complications [114,115]. Although its involvement in fibrosis is currently under investigation, existing evidence indicates that this cytokine possesses the capacity to stimulate inflammation, induce vascular injury, and activate fibroblasts [115]. Actually, vixarelimab is in a phase 2 clinical trial, aimed to evaluate changes in FVC from baseline to week 52 (NCT05785624). Axatilimab is an experimental monoclonal antibody designed to target the colony-stimulating factor-1 receptor (CSF-1R), a cell surface protein believed to control survival and functionality of monocytes and macrophages. Inhibiting signaling through the CSF-1 receptor is effective in reducing the number of disease-contributing macrophages and their monocyte precursors [116]. This reduction may play an essential role in mitigating the fibrotic disease processes associated with IPF. The ongoing phase 2 clinical trial is aiming to evaluate the efficacy and safety of axatilimab in patients with IPF (NCT06132256). EMT is an important pathogenic event in IPF, a phenomenon also observed in lung cancer, in which tumor cells express programmed death-ligand one (PD-L1) [117]. PD-L1 mediates lung EMT through Smad3 and β-catenin signaling pathways, contributing to fibrosis [118,119]. Based on this evidence, it is reasonable to suppose that immune checkpoint inhibitors such as atezolizumab may halt the progression of IPF, so a phase 1 clinical trial has already started to evaluate the safety and preliminary efficacy of atezolizumab (NCT05515627).

### 4.5. New Therapeutic Strategies: Stem Cells Therapy

In recent years, the use of embryonic stem cells for lung tissue regeneration has captured the attention of the scientific community. Stem cells exhibit notable anti-inflammatory and antifibrotic properties, positioning them as a potential therapy for fibrotic diseases, including IPF [120,121]. Several phase 1 clinical trials on stem cell therapy for IPF are currently ongoing, with the aim to assess the safety and feasibility of stem cell therapy (NCT05016817, NCT04262167, NCT01385644).

Mesenchymal stem cells (MSCs) are pluripotent cells with anti-inflammatory, immunosuppressive, and angiogenic functions, able to reduce extracellular matrix formation and collagen deposition. MSCs decrease the levels of TGF-β1 and TNF-α by producing prostaglandin E2 (PGE2) and hepatocyte growth factor [122]. Lung spheroid cells (LSC) comprise stem and support lung cells that can be cultured starting from lung tissue biopsies [123]. A study revealed that LSC treatment can attenuate and resolve bleomycin-induced fibrosis by reconstructing the normal alveolar structure, reducing collagen accumulation and myofibroblast proliferation [124]. When administered intravenously in a murine model of pulmonary fibrosis, lung spheroid cells demonstrated potent regenerative properties [125]. Some studies have shown encouraging results, with a significant improvement in FVC compared to the placebo group in patients receiving stem cell therapy [126,127,128]. Therefore, the use of stem cells in treating pulmonary fibrosis can be considered a promising future therapeutic strategy.

### 4.6. New Therapeutic Strategies: Tyrosine Kinase Inhibitors

Protein kinases have been associated with the fibrogenic process mediated by growth factors like TGF-β [129]. Activation of tyrosine kinases, particularly receptor tyrosine kinases (RTKs), results in the phosphorylation of tyrosine residues on target proteins, initiating cascades of intracellular signaling events. Dysregulation of tyrosine kinase activity was observed in various cell types within the lungs of IPF patients, including fibroblasts, epithelial cells, and inflammatory cells. This may contribute to the initiation and perpetuation of pro-fibrotic processes such as excessive ECM deposition and differentiation of fibroblasts into myofibroblasts. Several growth factor receptors and cytokine receptors, acting either as tyrosine kinases or able to activate tyrosine kinases upon ligand binding, are involved in the pathogenesis of IPF. Notably, receptors for PDGF, FGF, and epidermal growth factor (EGF) activate tyrosine kinases and are associated with fibrotic responses [130,131]. In addition to RTKs, non-receptor tyrosine kinases, including members of the Src family, also contribute to the pathogenesis of lung fibrosis [132]. These kinases are activated by various extracellular signals and mediate downstream effects that promote fibrosis, including cell proliferation, migration, and ECM remodeling. Tyrosine kinase inhibitors are currently utilized in the treatment of IPF for the selective inhibition of fibroblasts [130]. Nintedanib is an intracellular antagonist that selectively inhibits a range of tyrosine kinases, including the receptors for VEGF, FGF and PDGF [133]. Currently, it stands as one of the two approved antifibrotic treatments for IPF [1,51,134]. Understanding the specific tyrosine kinase pathways involved in IPF may provide additional potential targets for therapeutic intervention. Inhibitors targeting these kinases are currently explored as potential treatments to modulate aberrant signaling cascades and mitigate the fibrotic processes associated with IPF [57]. Saracatinib is a selective Src kinase inhibitor originally developed for oncological indications. Considering that Src-dependent processes also regulate myofibroblast differentiation and fibrogenic gene expression, a phase 1b/2a clinical trial has started to evaluate the use of saracatinib in the treatment of IPF (NCT04598919) [135,136].

The Janus kinase/signal transducer and activator of transcription (JAK/STAT) molecular pathway becomes activated in response to the interplay of a diverse array of profibrotic/pro-inflammatory cytokines and growth factors that are overexpressed in IPF, including PDGF, TGF-β1, and FGF [136,137]. These factors trigger JAK/STAT activation through both the canonical and non-canonical pathways, highlighting the relevant involvement of JAK/STAT in the fibrogenic process. Based on these findings, several clinical trials have addressed the evaluation of safety and efficacy of different drugs sharing the ability to block the JAK/STAT pathway (NCT05671835, NCT04312594).

## 5. Discussion

In recent years, significant progress has been made in the understanding of the pathobiology of IPF; however, these advancements are not fully satisfactory, and the approved drugs are still not curative. They may slow the decline of FVC, increasing survival, but are not able to block or reverse lung damage [138]. The primary goals of IPF patient management remain symptom alleviation, improvement in quality of life, and preservation of lung function over time. Even so, the identification of new potential therapeutic targets has been challenging, and the translation of these advances into drug development has been largely unproductive. It is important at this point to explore the possible pitfalls that determined the failure of several clinical trials, even in advanced phases. Most preclinical studies are performed on animals with experimentally induced fibrosis, keeping in mind that pharmacologically induced animal models of pulmonary fibrosis do not precisely reproduce the histological and pathophysiological patterns of IPF. Therefore, there is a need to identify animal models that more closely resemble IPF. Some clinical trials were prematurely terminated due to failure to reach the primary endpoint [5]. A possible explanation for this phenomenon may be the relative short observation period of 24 weeks for a disease characterized by an unpredictable course with phases of functional stability alternating with more rapid clinical worsening. This may also increase, particularly in phase 2 studies, the risk of excluding potentially promising molecules due to the incorrect selection of primary endpoints.

As regards endpoints of clinical trials, several studies have unequivocally demonstrated how FVC represents the main mortality predictor in IPF [139,140,141]. The role of FVC is further confirmed by its importance for the definition of PF-ILD [142]. On the other hand, a recent new point of view is considering replacing FVC as the primary endpoint with composite endpoints that include “feels, functions, survives” measures, with FVC as one of the components [143]. In recent years, several trials have initially given importance to the 6-min walking test (e.g., NCT04552899, NCT04396756) that was gradually abandoned due to the variability of the test within trials [144]. In addition, IPF still suffers due to late diagnoses often made in patients with very low DLCO values, between 25 and 30% [145]. Consequently, the trend of the latest clinical trials is to “lower the bar” to make patient participation more accessible. In considering the quest for the most accurate endpoint, it becomes pertinent to assess the real potential of composite endpoints. These endpoints, allowing the inclusion of multiple significant domains within a single predetermined endpoint, confer numerous advantages [146]. They facilitate a more comprehensive portrayal of a drug’s effects, predicting a range of events such as categorical alterations in FVC or the 6-min walking test distance, occurrences of respiratory hospitalization, changes in functional classification, transplant, or mortality.

It is known that not all IPF patients have the same course of the disease, with some being relatively “long survivors” and others with a more rapid progression of the disease possibly marked by exacerbations and in some cases affected by the presence of relevant comorbidities [147]. This makes IPF patients an extremely heterogeneous group, where each patient may have an individual response to the explored new drug. This individual response may be associated with different pathophysiological mechanisms, including the activation of specific pathways. The use of composite endpoints among patients enrolled in clinical trials may reduce this heterogeneity avoiding the fragmentation of these patients in small subgroups. As the understanding of the molecular mechanisms underlying IPF improves, it may be possible to devise personalized treatment strategies targeting specific pathways or genetic factors in individual patients.

Given the recognized role of TGF-beta in the pathogenesis of IPF, it is plausible that two drugs with complementary mechanisms may be more effective. A pharmacological treatment involving the use of multiple drugs should be taken into consideration. The INJOURNEY study evaluated the add-on therapy between pirfenidone and nintedanib with promising results in terms of safety and some preliminary insight into efficacy [148]. Unfortunately, this study was not followed by a larger trial specifically powered for the evaluation of efficacy. More recently, Bonella et al. highlighted that most experimental new drugs are evaluated as adjunctive therapy to the already approved antifibrotic therapies. Considering the intriguing possibility of targeting multiple coactivated profibrotic pathways, the optimal drug partners are those with complementary, alternative, or synergistic mechanisms of action compared to the standard of care [149]. In oncology, tailored and combined treatments represent a therapeutic keystone, always based on a careful evaluation of the individual patient characteristics, including a molecular and histopathological assessment. Over time, IPF was compared with cancer [97,150,151,152]. Both IPF and lung cancer are marked by epigenetic modifications, such as DNA methylation and histone modifications, that may alter gene expression without changing the DNA sequence. These epigenetic changes affect genes associated with cell cycle control, apoptosis, and ECM remodeling, processes known to be associated with both fibrosis and cancer [153,154]. Both diseases have limited treatment options and are associated with high mortality rates. However, the shared pathogenic pathways also present opportunities for novel therapeutic strategies [151]. Repositioning of drugs already used in oncology could be an interesting option for the treatment of IPF, taking advantage of the multiple shared pathogenic mechanisms between the two diseases. Nintedanib, originally used for the treatment of lung cancer, is the practical demonstration of this concept. In oncology, artificial intelligence (AI), has demonstrated effectiveness in exploring genotypes and phenotypes for early diagnosis, screening, and personalized treatment regimens based on genetic-oriented features [155]. In respiratory diseases, AI and machine learning is mainly used to evaluate lung cancer and pulmonary fibrosis CT scan images [156]. More recently, for the first time in respiratory medicine, a new molecule developed entirely from AI, targeting alfa-SMA protein, will be tested in a phase 3 clinical trial for IPF treatment (NCT05938920) [157]. This AI-designed molecule may represent a breakthrough, as it demonstrates the potential of AI in accelerating drug discovery processes by predicting the structure and function of novel molecules. These interesting results are likely indicating a new different approach for the identification of new molecules to treat IPF, suggesting that AI could play a pivotal role in the future of personalized medicine and targeted therapy development. Additionally, the integration of AI in respiratory medicine could lead to more personalized and effective treatment strategies, tailored to the specific genetic and molecular profiles of individual patients, thereby enhancing treatment outcomes and reducing adverse effects. These interesting results are likely indicating a new different approach for the identification of new molecules to treat IPF. Moreover, the role of genetic factors in the pathogenesis of IPF should not be underestimated. Over the years, significant progress was made in the potential application of gene therapy for the treatment of pulmonary fibrosis in vivo. Indeed, gene therapy may offer new and promising avenues to mitigate a broad range of processes involved in the development of fibrosis [158].

## 6. Conclusions and Future Perspective

Despite evident progress in understanding the pathophysiology of IPF and the increasing number of studies searching for new molecules capable of slowing or halting the progression of the disease, IPF still remains a clinical unsolved problem. The therapies currently available do not offer curative possibilities but aim to slow down the progression of the disease, still characterized by an unfavorable prognosis. This review provides insights into potential therapeutic targets and a more comprehensive evaluation of the entire pathogenetic process of IPF, which involves the activation of synchronous, multiple pathogenic pathways. A better understanding of the degree of activation of these pathways could lead to new approaches in the treatment of IPF, with targeted therapy adapted to the individual characteristics of each patient. Clinical trials must be based on robust, high-quality preclinical and clinical data, with precise and agreed-upon endpoints. There are many promising drugs in development, resulting from a continuous, yet insufficient investment in IPF research. Furthermore, our understanding of the pathogenesis of IPF remains limited, and it is imperative to direct our efforts towards in-depth insight into the mechanisms that underlie this complex disease and their impact on clinical phenotypes.

## Figures and Tables

**Table 1:** An overview of clinical trials of in IPF.

Molecule	Mechanism of Action	ClinicalTrial.gov Identifier	Phase
Anlotinib	Inhibiting kinases	NCT05828953	2–3
Treprostinil	Prostacyclin receptor agonist	NCT04708782, NCT05255991	3
BI 1015550	PDE-4B inhibitor	NCT05321069	3
LTI-03	Inhibiting ECM production	NCT05954988	1
BMS-986278	LPA1 antagonist	NCT06003426	3
HZN-825	LPAR1 antagonist	NCT05032066	2
XFB-19	Inhibiting C/EBPβ	NCT05361733	1
ARO-MMP7	RNA interference	NCT05537025	1–2a
Venetoclax	Inhibiting Bcl-2	NCT05976217	1
HuL001	Anti-ENO1 mAb	NCT04540770	1
Sufenidone	Inhibiting TGF-β1	NCT06125327	2–3
Deupirfenidone	Inhibiting TGF-β1	NCT05321420	2
Yifenidone	Inhibiting TGF-β1	NCT05060822	2
SRN-001	RNA interference	NCT05984992	1
Bexotegrast	Inhibiting integrins	NCT06097260	2
Artesunate	Downregulating TGF-β1	NCT05988463	1
Vixarelimab	Anti-OSMR mAb	NCT05785624	2
Axatilimab	Anti-CSF-1R mAb	NCT06132256	2
Atezolizumab	Anti-PD-L1 mAb	NCT05515627	1
Umbilical cord derived MSCs	Immunomodulation, anti-fibrosis, and tissue regeneration	NCT05016817	1
Lung Spheroid Stem Cells		NCT04262167	1
Placental MSCs		NCT01385644	1
Saracatinib	Inhibiting Src kinase	NCT04598919	1b/2a
TTI-101	Inhibiting STAT3	NCT05671835	2
Jaktinib Hydrochloride	Inhibiting JAK1/2	NCT04312594	2

Legend: PDE-4B: phosphodiesterase-4B inhibitor; ECM: extracellular matrix; LPA1: lysophosphatidic acid; LPAR1: lysophosphatidic acid receptor 1; C/EBPβ: CCAAT/enhancer-binding protein beta; ENO1: alpha-enolase; mAb: monoclonal antibody; TGF-β1: transforming growth factor-β 1; OSMR: oncostatin M receptor; CSF-1R: colony-stimulating factor-1 receptor; PD-L1: programmed death—ligand 1; STAT3: signal transducer and activator of transcription 3; JAK1/2: Janus kinase 1/2 inhibitor.

## Data Availability

Not applicable.
